# Dopant-Tunable Ultrathin Transparent Conductive Oxides for Efficient Energy Conversion Devices

**DOI:** 10.1007/s40820-021-00735-y

**Published:** 2021-10-16

**Authors:** Dae Yun Kang, Bo-Hyun Kim, Tae Ho Lee, Jae Won Shim, Sungmin Kim, Ha-Jun Sung, Kee Joo Chang, Tae Geun Kim

**Affiliations:** 1 School of Electrical Engineering, Korea University, Seoul, 02841 Republic of Korea; 2 Department of Advanced Materials Engineering, Kongju National University, Cheonan, 31080 Republic of Korea; 3 Department of Physics, Korea Advanced Institute of Science and Technology, Daejeon, 34141 Republic of Korea

**Keywords:** Transparent conductive oxide, Metal implantation, High transparency, Low sheet resistance, Work function

## Abstract

Ultrathin film-based transparent conductive oxides (TCOs) with a broad work function (WF) tunability are highly demanded for efficient energy conversion devices. However, reducing the film thickness below 50 nm is limited due to rapidly increasing resistance; furthermore, introducing dopants into TCOs such as indium tin oxide (ITO) to reduce the resistance decreases the transparency due to a trade-off between the two quantities. Herein, we demonstrate dopant-tunable ultrathin (≤ 50 nm) TCOs fabricated via electric field-driven metal implantation (*m*-TCOs; *m* = Ni, Ag, and Cu) without compromising their innate electrical and optical properties. The *m*-TCOs exhibit a broad WF variation (0.97 eV), high transmittance in the UV to visible range (89–93% at 365 nm), and low sheet resistance (30–60 Ω cm^−2^). Experimental and theoretical analyses show that interstitial metal atoms mainly affect the change in the WF without substantial losses in optical transparency. The *m*-ITOs are employed as anode or cathode electrodes for organic light-emitting diodes (LEDs), inorganic UV LEDs, and organic photovoltaics for their universal use, leading to outstanding performances, even without hole injection layer for OLED through the WF-tailored *Ni*-ITO. These results verify the proposed *m*-TCOs enable effective carrier transport and light extraction beyond the limits of traditional TCOs.

**Supplementary Information:**

The online version contains supplementary material available at 10.1007/s40820-021-00735-y.

## Highlights

- Dopant-tunable transparent conductive oxide (≤ 50 nm) fabricated via electric-field-driven metal implantation (m-TCOs; m= Ni, Ag, and Cu) is demonstrated.
- The m-TCOs exhibit ultrahigh transparency, low sheet resistance, and broad work function tunability, leading to outstanding performance in various optoelectronic devices.
- The work function change is attributed to the interstitial metal atoms that provide the empty d-orbital, resulting in the shift of the Fermi level.

**Supplementary Information:**

The online version contains supplementary material available at 10.1007/s40820-021-00735-y.

## Graphical abstract

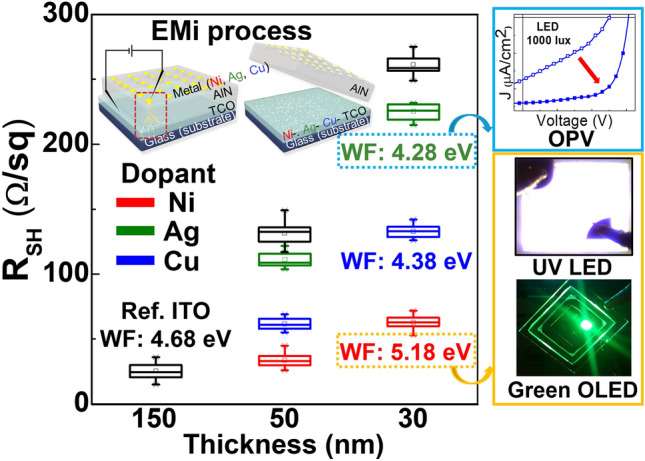

## Introduction

Modern energy conversion devices, including organic/inorganic solar cells [1] and light-emitting diodes [2], require innovative thin transparent electrodes with high electrical conductivity and optical transparency. To satisfy the growing demand for advanced transparent electrodes, transparent conductive oxides (TCOs) such as indium tin oxide (ITO) [3], aluminum-doped ZnO (AZO) [4], and fluorine-doped SnO (FTO) [5] have been developed, along with various types of transparent electrodes such as metal nanomesh [6], metal nanowire [7], conductive polymer [8], and graphene [9] over the past several decades. However, most conventional TCOs are *n*-type owing to localized oxygen, which impedes band alignment and charge balance when they are used as *p*-electrodes of such devices [10], thereby resulting in low quantum efficiency. Moreover, as modern optoelectronic devices are becoming thinner and more flexible, TCOs need to be thinner while maintaining adequate electrical conductivity. However, electrical resistivity and optical transparency are mutually contradictory with respect to the TCO thickness; therefore, reducing film thickness below 50 nm has been limited due to rapidly increasing sheet resistance (*R*_SH_) [11].

In addition, the work function (WF) of TCOs is important for balancing charge injection via the band alignment of devices. The WF of TCOs can be fine-tuned by adjusting the oxygen content [12] and modifying the surface using self-assembled molecules and polymers [13] or multilayer films [14]. However, applying these methods to practical devices is difficult owing to the strict requirements for high transparency, low *R*_SH_, and WF alignment. Considerable effort has been exerted for developing *p*-type TCOs, and notably, the hybridization of oxygen and Cu ions has triggered the development of various Cu-based oxides such as CuMO_2_ (e.g., M = Al, Cr, and In) [15]. Presently, the highly promising p-type TCOs are CuAlO_2_ and MgCr_2_O_3_ [16, 17]; however, the metastability of Mg and Cu ions and the complexity of compound oxides remain technical challenges in practical applications. Moreover, the low conductivity and/or the loss of visible-light transparency of Mg/Cu-TCOs are obstacles that need to overcome for further improvements in optoelectronics.

One promising way to solve these problems is to dope the surfaces of electrodes with donors or acceptors, through co-sputtering [18], ion implantation [19], or optical excitation [20], during the fabrication process. However, with conventional doping methods, we cannot independently control the electrical and optical properties of TCOs. For instance, the transmittance of TCOs decreases as the doping concentration increases, or vice versa [21]. Moreover, the doping process by co-sputtering is known to be quite sensitive to the material composition and applied power [18], whereas the other two methods require a costly external high-power source for dopant injection [19, 20]. Therefore, it is important to find a way of tailoring the WF of ultrathin TCOs without compromising their innate electrical and optical properties.

In this study, we devised an electric field-driven metal implantation (EMi) method that can lower the *R*_SH_ and properly tailor the WF of thin TCO films without transmission losses. Notably, EMi is a unique doping method that allows the independent control of the electrical and optical properties of TCOs, via the diffusion of electrochemically active metal dopants (e.g., Ni, Ag, and Cu) into TCOs under an electric field [22]. This method has advantages of simple, cost-effective, and annealing-free processing, as well as selective doping capability, over conventional doping methods. Herein, we present ultrathin (≤ 50 nm) TCOs doped by metals using the EMi method (*m*-TCOs; *m* = Ni, Ag, and Cu). The EMi metals doped on the surface of TCOs (ITO, AZO, and FTO) are examined using spectroscopic methods to show that doped metals are embedded as metallic and ionic states into the epidermal layers of TCOs. The metal doping by the EMi method induces a sharply decreased R_SH_, but the transmittance of TCOs is not critically deteriorated. Furthermore, the WF of TCOs is varied according to the type of metal dopant. The underlying mechanism of varying WF is theoretically studied, showing that interstitial metals rather than substitutional ones have more dominant effects on the shift of the Fermi level. Based on the dopant-tunable ultrathin TCOs, we demonstrate outstanding performances of organic light-emitting diodes (OLEDs), inorganic UV LEDs, and organic photovoltaics (OPVs).

## Experimental Section

### Transparent Conductive Oxides

ITO films were deposited using an electron-beam (E-beam) evaporation system (KVE-E2003L, Korea Vacuum Tech) with an ITO 4 N source (VTM, Republic of Korea). The samples were annealed using rapid thermal processes (RTP, RTA-150H SP1, KVE-E2003L) at 450 °C for 60 s. AZO and FTO films were then deposited using a radio frequency (RF) magnetron sputtering system (KVS-4000, Korea Vacuum Tech) with an Al-doped ZnO (2:98) 2 N target (RND KOREA, Republic of Korea) and an FTO (1:9) 2 N target (RND KOREA, Republic of Korea). A 10-nm AlN layer was also deposited using a radio frequency (RF) magnetron sputtering system and annealed using RTP in an N_2_ gas environment at 450 °C for 30 s for recrystallization. Next, a 50-nm-thick circular metal (Ni, Ag, Cu) pad for the EMi process was deposited using an RF magnetron sputtering system with a metal (Ni, Ag, Cu) 3 N target (RND KOREA, Republic of Korea) through photolithography using the lift-off process with an AZ 5214 negative photoresist and CD30 developer.

### Electric field-driven Metal Implantation

The EMi process for metal implantation into the TCOs was conducted by applying a DC bias across the AlN layer between the top metal pad and TCO bottom film. The metal pad deposited for the EMi process had a diameter of 50 μm; a 10 μm gap was maintained between each dot. The size of metal dot and the gap should be decreased for better current injection and spreading (lateral distribution) effects in TCOs; however, this occasion requires more time to scan the surface of TCOs for the EMi process. Therefore, they should be properly optimized by considering the efficiency and processing time of the EMi. A two-point auto-probing system (PS-4A2P, Modusystems) was used for the EMi process. After the EMi process, the metal pad and AlN layers were removed using a wet etching process with a nichrome etchant (Sigma-Aldrich) and AZ400K (AZ Electronic Materials), respectively.

### Material Characterization

The *R*_SH_, carrier concentration, and hole mobility were measured using a hole measurement system (HMS3500/HT55T5, ECOPIA). The optical transmittance of the TCOs was measured by using a UV/VIS spectrometer (Lambda 35, PerkinElmer). The WF of the TCOs was measured using a KP system (Kelvin Control 07 with a probe diameter of approximately 3 mm, Besocke Delta Phi GmbH) in Faraday cages. Film characteristics such as conductivity, optical transmittance, and WF values of TCOs were measured after the EMi process without performing any other treatments. Transmission electron microscopy (TEM, 200F, JEOL) and X-ray photoelectron spectroscopy (XPS, ULVAC-PHI, PHI-X-tool) were used to analyze the chemical and crystalline structures of the TCOs.

### Density Functional Theory Calculation

The first-principles density functional calculation was conducted using the GGA for the exchange–correlation potential [23] and the projector-augmented wave potentials [24], as implemented in the Vienna Ab initio Simulation Package code [25]. We selected a slab geometry wherein In-terminated ITO layers are in contact with a vacuum region with an Sn/In ratio of 0.11. The on-site Coulomb correlation (*U*) was included because the positions of the metal d bands are overestimated by the GGA functional, where the chosen parameter values were *U* = 5, 2.5, and 7.0 eV for Ni 3*d*, Sn 4*d*, and In 4*d*, respectively [26]. Furthermore, to obtain reliable band gaps, we employed the hybrid functional form proposed by Heyd, Scuseria, and Ernzerhof for the exchange–correlation potential with a screening parameter of ω = 0.2 Å^−1^ and a mixing fraction of α = 0.25 [27].

### Device Fabrication

#### OLEDs

4,40,400-tris(N-carbazolyl)-triphenylamine (TCTA)/2,2,2-(1,3,5-benzenetriyl) tris-(1-phenyl-1Hbenzimidazole) (TPBi)-based green phosphorescent OLEDs were fabricated. A 50-nm N,N-di (naphtha-lene-1-yl)-N,N-diphenylbenzidine hole transport layer, a 10-nm TCTA exciton-blocking layer (EBL), a 20-nm co-deposited TCTA/TPBi layer with a 3:7 ratio doped using a 12 wt% Ir(ppy)_3_ emission layer, a 40-nm TPBi electron transport layer, a 1.5-nm LiF electron injection layer, and a 100-nm Al top cathode were deposited using a thermal evaporator (SUNIC System, Republic of Korea) in a vacuum chamber at 2 × 10^–7^ Torr. Finally, the samples were encapsulated using a dispenser (Shot Mini 200S-3A, MUSASHI) with epoxy and a 1.6 cm × 1.6 cm × 0.7 T encapsulation glass in an N_2_-filled glove box. The size of each active area was 3 × 3 mm^2^. The *J–L–V* curves of the OLEDs were measured using a luminance meter (CS-100A, Konica Minolta) coupled with a Keithley 2400 voltage and current source meter. The EQE and EL spectral characteristics were measured using a spectroradiometer (CS-2000, Konica Minolta) coupled with a Keithley 2400 voltage and current source meter.

#### UV LEDs

*p*-Al_0.1_Ga_0.9_ N-terminated 365-nm UV LEDs were fabricated. A typical LED structure comprises a 5-μm undoped AlN buffer layer grown on sapphire, followed by a 1-μm Si-doped n-Al_0_._2_Ga_0_._8_ N layer, 2-μm Si-doped n-Al_0_._1_Ga_0_._9_ N layer, AlGaInN/InGaN-based 75-nm four-pair undoped multiple quantum well region, 10-nm Mg-doped *p*-Al_0.1_Ga_0.9_ N layer, 20-nm Mg-doped *p*-Al_0.2_Ga_0.8_ N layer, and 150-nm Mg-doped (~ 10^17^ cm^−3^) *p*-Al_0.1_Ga_0.9_ N contact layer. First, standard photolithography and inductively coupled plasma reactive-ion etching were used to form isolated fan-shaped mesa structures for the *n*-type metal contact. TCOs were then deposited onto the isolated mesa pattern as a *p*-type transparent electrode, and the EMi process was applied. Finally, Cr/Ni/Au layers were deposited as *p*- and *n*-type metal electrodes using an e-beam evaporation system. The size of each chip was 390 × 390 μm^2^. We evaluated the device performance of each UV LED using an LED measurement system (PLATO, EtaMax Co., Ltd.) with a Keithley 2400 source measure unit.

#### OPVs

Inverted-type indoor OPVs consisting of *Ag*-ITO/ZnO/poly(3-hexylthiophene(P3HT):Indene-C60 bisadduct(ICBA)/MoO_X_/Ag were fabricated. A ZnO sol‒gel solution was prepared by mixing 1.24 g of zinc acetate dihydrate (Zn(CH_3_COO)_2_ 2H_2_O) and 0.5 g of ethanolamine in 2-methoxyethanol (10 g), all of which were obtained from Sigma-Aldrich (St. Louis, MO, USA). The solution was spin-coated onto the substrate at 4000 rpm for 60 s, followed by annealing at 200 °C for 30 min. A P3HT solution (4002E, Rieke Metals, Lincoln, NE, USA):ICBA (Luminescence Technology Corp., New Taipei City, Taiwan) was prepared in 1,2-dichlorobenzene (Aldrich, St. Louis, MO, USA) at a weight ratio of 1:1 (P3HT:ICBA) to achieve a total P3HT + ICBA concentration of 40 mg mL^−1^. The P3HT:ICBA solution was stirred at 70 °C overnight in a nitrogen-filled glove box before use. The samples were loaded into a vacuum thermal evaporation system, and a 10-nm layer of MoO_X_ was deposited through a shadow mask at 0.1–0.15 nm s^−1^ with a base pressure of ~ 1 × 10^−7^ Torr. Without breaking the vacuum, a 100-nm Ag layer was deposited through the previously used shadow mask at 0.1–0.2 nm s^−1^ with a base pressure of ~ 1 × 10^−7^ Torr. The *J–V* characteristics of the OPVs were measured using a source meter (Keithley 2401) coupled with a solar simulation program (K730, McScience Co., Ltd). An LED lamp emitting 1000 lx (McScience; I_L_ = 0.28 mW/cm) was used as the light source.

## Results and Discussion

### Fabrication of *m*-TCOs via Electric Field-Driven Metal Implantation

**Fig. 1:**
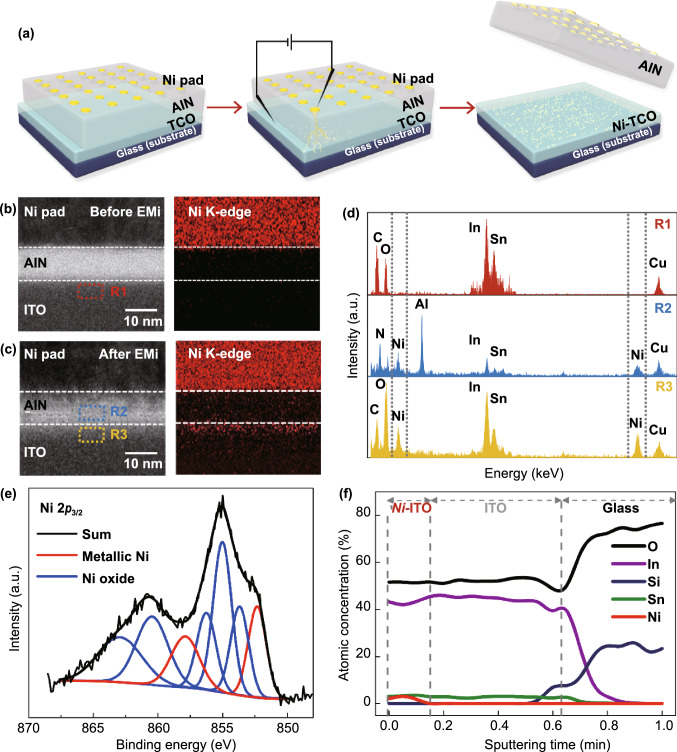
Metal implantation on the surface of TCOs: **a** Schematic of EMi process. HR-TEM images and Ni EDS mapping images **b** before and **c** after EMi. **d** EDS spectra measured at R1, R2, and R3 in Figs. 1b and 1c. **e** XPS core-level spectra of Ni 2*p* peaks before and after EMi. **f** Ni, Sn, Si, In, and O concentrations of the *Ni*-ITO films with respect to the depth from the ITO surface

Figure 1a shows a schematic of the EMi process carried out on a thin film of sequentially deposited TCO (ITO, AZO, or FTO), sacrificial buffer (AlN), and metal (Ni, Ag, or Cu). The sacrificial AlN layer serves not only to protect the TCOs from damage by electric shocks but also to provide a passage for metal ions coming from the metal pad to the TCO layer. When a sufficiently high electric field (> 2.1 MV cm^−1^) is applied on the metal pads, metal conduction pathways are instantaneously opened, similar to the lightening discharge channel in the AlN layer (Fig. 1a and S1a, b) [28]. The EMi voltage (which is the voltage required to produce conductive doping channels) varies with the buffer layers (Fig. S1c). In this study, the thickness of the TCO layer is set to 30–50 nm, which is much thinner than that of conventional TCOs (150–180 nm). The AlN buffer layer and metal pad are removed after conducting the EMi process. Figure 1b, c shows the high-resolution transmission electron microscopy (HR-TEM) and energy-dispersive X-ray spectroscopy (EDS) images before and after EMi using Ni on ITO. Before EMi, the interfaces between layers are clearly observed; further, Ni is not detected in the AlN and ITO layers (Fig. 1b). By contrast, after EMi, the interfaces between layers become vague, and Ni signals appear in both the AlN and ITO layers (Fig. 1c). The Ni signal observed at the epidermal layer of ITO is the strongest, and this signal decreases and disappears as the depth increases, suggesting that the doped metal ions are predominantly located within the depth of a few nanometers, although some could migrate deeply into the ITO. Figure 1d presents the EDS spectra measured at R1 in Fig. 1b (top spectrum), and R2 and R3 in Fig. 1c (middle and bottom spectra), respectively. Notably, the Ni signal clearly appears in the R3 region, whereas the Ni signal observed in R2 is relatively weaker, and there is no Ni signal in R1. This indicates that the high electric field drives metal ions to the epidermal layer of TCO through the AlN layer [29]. This phenomenon is observed in all EMi results obtained with different metals and TCOs, suggesting the proposed EMi method can be universally applied. This feature is distinguishable from normal doping occurring in semiconductors. The effective penetration depth of EMi, which corresponds to the projection range of ion implantation, can be controlled based on the types of buffer materials and their thicknesses, as well as the electric field magnitude. In this experiment, we used 10-nm AlN as a buffer layer for all *m*-EMi owing to its lower EMi voltage than other materials such as Al_2_O_3_, Si_3_N_4_, and SiO_2_ (Fig. S1c) and easy removal by a solvent (CD30 developer). The surface morphology of the TCOs does not critically change after EMi and the removal of the AlN layer (Fig. S2). The wide-scan spectra of X-ray photoelectron spectroscopy (XPS) show that Ni 2*p* peaks only appear at the ITO surface after EMi (Fig. S3a), consistent with the EDS results of TEM. From the spectral decomposition of the Ni 2*p*_3/2_ peak (Fig. 1e), the coexistence of metallic nickel (Ni^0^; 852.4 and 858.1 eV) and nickel oxides, including nickel hydroxide and its compounds (853.6, 855.0, 856.4, 860.8, and 863.5 eV), is revealed [30]. The Ni atom has a smaller atomic radius, greater electron affinity, and higher electron negativity than those of the In atom. In addition, the dissociation energy (396 kJ mol^−1^) of the Ni–O bond is higher than that (320.1 kJ mol^−1^) of the In-O bond [18, 31]. Therefore, instead of In-O bond formation, Ni–O bonds are formed by the Ni atoms implanted into the ITO, which is reflected by the variation in the oxygen binding energy, while the change in the In and Sn binding energies is negligible (Fig. S3b–d). Figure 1f shows the variation of atomic concentration, with respect to the etching (= sputtering) time, based on an XPS depth profile. At an etching time of less than 8 s (depth < 10 nm), the atomic percentage of Ni is observed to be 2.5–3 at%, and that of In decreases from ~ 45 to 42 at%, while that of Sn is constantly maintained. As the etching time further increases, Ni disappears and In is saturated until the interface between the ITO and glass substrate is approached. These analyses firmly indicate that the implanted Ni exists as interstitial atoms and substitutes for In within 10 nm from the ITO surface.

### Optical and Electrical Properties of *m*-TCOs

**Fig. 2:**
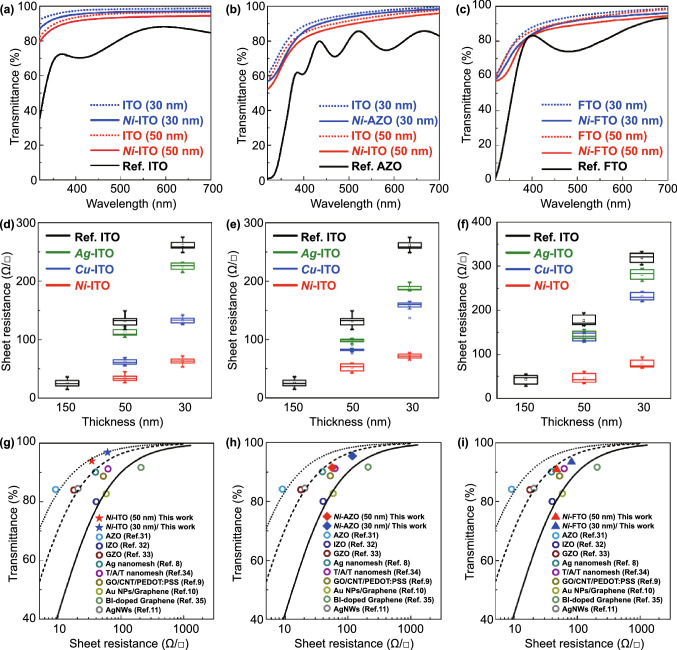
Transmittance and *R*_SH_ of *m*-TCOs: **a–c** Comparison of UV–visible transparency spectra of **a** *Ni*-ITO, **b** *Ni*-AZO, and **c** *Ni*-FTO. **d–f** *R*_SH_ of **d** *m*-ITO, **e** *m*-AZO, and **f** FTO depending on the layer thickness. *m* is Ni (red), Ag (green), and Cu (blue). The black is the reference of each TCO. Transmittance (at 550 nm) as a function of *R*_SH_ of **g** *m*-ITO (solid star symbols), **h** *m*-AZO (solid rhombus symbols), and **i** *m*-FTO (solid triangle symbols) samples alongside other reference samples. A series of solid and dotted lines represent curves calculated using the Tinkham equation corresponding to σOp/σDC values of 43 (solid line; minimum industry standard), 100 (dash line), and 200 (dotted line)

Thin-film electrodes must have two essential properties—transparency and electrical conductivity; however, these properties are contradictory to each other owing to their inverse proportional relationship. To investigate the effect of EMi on this inverse relation, we examined the transmittance and *R*_SH_ of the 30- and 50-nm TCOs after the EMi process and performed a comparison with the TCOs before EMi (Fig. 2). In Fig. 2a–c, the transmittance of (a) ITO, (b) AZO, and (c) FTO explicitly increases as the thickness decreases, regardless of the EMi process. After *Ni*-EMi, all TCOs show a high transmittance (> 85%) over the entire visible range (400–700 nm), which is lower by less than 2% compared to those of the TCOs before EMi. In particular, the transmittance of *Ni*-ITO is maintained to be over 80% in the UV range (320–400 nm) (Fig. 2a), while that of Ref. ITO (commercially purchased, 150 nm) sharply decreases to 35% at 320 nm. Similarly, *Ag*- and *Cu*-ITOs show a high transmittance (> 80%) in the entire visible range (Fig. S4). Notably, regardless of metal dopants, *m*-ITO commonly shows a high transmittance in the UV to visible range, indicating that it is a prospective electrode for UV optoelectronic devices.

Furthermore, the *R*_SH_ of *m*-TCOs was measured as a function of layer thickness depending on the implanted metals, using the four-point probe method (Figs. 2d–f and S5). The *R*_SH_ of TCOs before EMi is inversely proportional to the film thickness, as expected. However, after EMi, *m*-TCOs show an explicit decrease in *R*_SH_ compared to those of TCOs before EMi, although the *R*_SH_ of *m*-TCOs increases as the film thickness decreases. Moreover, among three metals (Cu, Ag, and Ni), Ni is the most effective dopant for reducing the *R*_SH_ by as much as ~ 77% (ITO), ~ 64% (AZO), and ~ 74% (FTO), respectively. The *R*_SH_ of *Ni*-ITO, in particular, is as low as 61 ± 10 Ω/□ (30 nm) and 34 ± 11 Ω/□ (50 nm), which is of the same order as the *R*_SH_ of Ref. ITO (150 nm), namely 26 ± 11 Ω/□ (Fig. 2d). For *m*-AZO and *m*-FTO, the lowest *R*_SH_ is 87 ± 18 and 47 ± 15 Ω/□ for *Ni*-AZO (50 nm) and *Ni*-FTO (50 nm), respectively (Fig. 2e, f). The *R*_SH_, resistivity, and transmittance of the *Ni-*TCOs at 550 nm are listed in Table S1. To further investigate the electrical property of the *Ni*-TCOs, we measured the carrier density and hole mobility (Table S2). The carrier density of *Ni*-TCOs (Ref. TCOs) is of the order of 10^21^ (10^20^) cm^3^, increasing up to ~ 104% in *Ni*-ITO (30 nm). Moreover, the hole mobility of *Ni*-TCOs shows an almost twofold increment, with the largest value of 26 cm^2^ V^−1^ s^−1^ at *Ni*-ITO (30 nm) compared to those before EMi.

Overall, *m*-TCOs are less affected by the inverse relation between transmittance and *R*_SH,_ with respect to the film thickness; further, *Ni*-EMi is the most effective in tailoring opto-electrical properties of TCOs. This is because the coexistence of metallic Ni and Ni oxide gives rise to some light scattering sites, thereby inducing the enhancement of the light outcoupling intensity (Fig. S6a). In addition, when Ni^2+^ ions are added to In_2_O_3_, axial orbitals arising from the splitting of d-orbital (*e*_g_ levels) of Ni^2+^ impurities are known to exist below the conduction band of In_2_O and easily undergo *sp-d* hybridization with a relatively lower energy than that in the case of Ag and Cu [32]. In this regard, the *e*_g_ levels of Ni^2+^ impurities are relatively higher than those of Ag and Cu; therefore, the small reduction in the band gap energy and the expansion of conduction band-like states in *Ni*-ITO may be able to facilitate the charge transport. Furthermore, Ni has a smaller ionic diameter than those of Ag and Cu. This could possibly contribute to the reduction of carrier scattering in *Ni*-ITO, compared to *Ag*- and *Cu*-ITO, via the suppression of oxygen defects [33]. These multiplicative factors increase the electrical performance of *Ni*-ITO drastically compared to those of *Ag*- and *Cu*-ITO. Nevertheless, these doped Ni atoms do not significantly change the structural and morphological properties of TCOs, leading to a trivial difference in angle-dependent light outcoupling efficiency of the fabricated device (Fig. S6b).

For a more quantitative analysis of the relationship between *R*_SH_ and transmittance, we calculated the figure of merit (FOM). In general, the *R*_SH_ and transmittance values of TCOs can be expressed with the Tinkham formula given in Eq. (1):

1 $${\text{T}}\left( {\uplambda } \right) = \left( {1 + \frac{188.5}{{R_{s} }}\frac{{{\upsigma }_{Op} \left( {\uplambda } \right)}}{{{\upsigma }_{DC} }}} \right)^{ - 2}$$

where σ_Op_(λ) is the optical conductivity (here, at 550 nm) and σ_DC_ is the DC conductivity of the film [34]. In this formula, the ratio of σ_DC_/σ_Op_ can be considered as the FOM for the TCOs, as it can provide an intuitive understanding of the relation between the *R*_SH_ and transmittance of the TCOs. From an industrial perspective, 43.85 (*R*_SH_ ≤ 100 Ω/□ and transmittance at 550 nm ≥ 90%) is commonly regarded as the required minimum FOM value [35]. Figure 2g shows the transmittance (at 550 nm) versus *R*_SH_ of the 30- and 50-nm *Ni*-ITO (solid star symbols) in comparison with those of the various types of transparent electrodes reported in the literature [6–9, 36–40]. All *m*-TCOs (ITO, AZO, FTO) studied here yielded FOM values ranging from 79 to 194 (Fig. 2g–i), which exceed the minimum standard σ_DC_/σ_Op_ value required in the industry. Furthermore, the FOM values of the proposed *m*-TCOs are much greater than those obtained in other studies.

### Work Function Engineering of *m*-TCOs

**Fig. 3:**
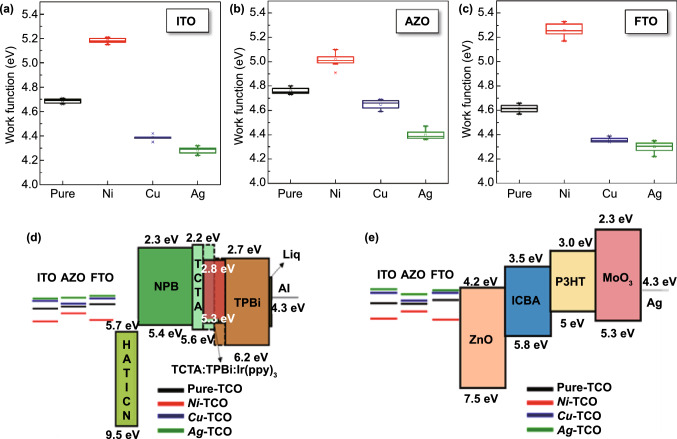
Work function of *m*-TCOs: **a** *m*-ITO, **b** *m*-AZO, and **c** *m*-FTO. Schematic energy band diagram of the components that comprise the **d** OLED and **e** OPV with the various EMi applied electrodes (Ref. TCO and *m*-TCO)

Till date, the direct transition of *n*-type TCOs to *p*-type TCOs through surface modification by metal implantation has not been reported, except for surface modification using self-assembled molecules or co-deposition of metals [12–14]. To investigate the possibility of such a transition via EMi, we probed the WF of *m*-TCOs using an independent Kelvin probe (KP) microscope and performed a double check using UV photoelectron spectroscopy (Fig. 3 and Table S3). After *Ni-*EMi, the WF of *Ni*-ITO increases up to 5.18 eV (from 4.68 eV before EMi), whereas *Cu*- and *Ag*-EMi on ITO decrease the WF to 4.38 and 4.28 eV, respectively (Fig. 3a). The variations among the WFs of the *m*-ITOs reach up to 0.9 eV, which is remarkable compared to the previous reports [5, 10, 12]. Moreover, the WF values of *Ni*-, *Cu*-, and *Ag*-ITO are similar to those of pure Ni (~ 5.17 eV), Cu (~ 4.39 eV), and Ag (~ 4.26 eV), as proven by the KP results, while they are lower than those of nickel oxide (> 5.2 eV) [41], copper oxide (> 5.2 eV) [42], and silver oxide (> 5.26 eV) [43]. This trend is similarly observed in *m*-AZO (Fig. 3b) and *m*-FTO (Fig. 3c) with variations depending on TCO types. The WF variation in *m*-AZO is 0.62 eV, with a minimum WF value of 4.39 eV and a maximum of 5.01 eV, while in *m*-FTO, it is 0.95 eV, with minimum and maximum WF values of 4.30 eV (*Ag*-FTO) and 5.25 eV (*Ni*-FTO), respectively. The WF change of materials (i.e., *Ni*-ITO, for example) is closely associated with the doping concentration of the Ni atoms exposed at the surface after the vacancy sites in ITO are occupied, as well as the formation of Ni–O bonds at the surface. As stated previously, Ni atoms injected into ITO can more easily produce a Ni–O bond (compared to the formation of an In-O bond) owing to the smaller atomic radius, greater electron affinity, and higher electron negativity of Ni atoms than those of In atoms. Further, Ni–O bond has a higher dissociation energy (396 kJ mol^−1^) than In-O bond (320.1 kJ mol^−1^) [18, 31]. Therefore, the size, electron affinity, and electron negativity of doping elements can influence the WF change of materials. This analogy also applies to other *m*-TCOs. In addition, the WF of *m*-TCOs is affected by the thickness of the TCO layer; however, the variation due to this thickness is not very high (< 0.1 eV); this is because of the low doping concentration (i.e., 2.5%–3% for Ni) in the epidermal layer (within a few nanometers) of TCOs (Table S3). With this tunable WF of TCOs, the energy bands can be well aligned in various energy conversion devices, including LEDs and OPVs. Figure 3d, e schematically shows the electronic band diagrams of OLED and OPV devices, respectively, in which the OLED uses *m*-TCOs as the anode and an OPV as the cathode. For the fabrication of practical devices, *Ni*-TCOs will be appropriate as anode electrodes because of the raised WF value (Fig. 3d), whereas *Ag*-TCOs have an advantage as cathodes in OPVs due to the lowered WF value (Fig. 3e). Details on the practical devices using *m*-TCOs will be discussed later. On the other hand, we investigated the WF changes of the *Ni*-ITO and Ref. ITO over time to figure out the long-term stability of the TCO after EMi (Fig. S7). The result indicates that well-tailored WF values of *Ni*-ITO are stably maintained over time.

### Theoretical Analysis for the Mechanism of Dopant-tunable *m*-TCOs

**Fig. 4:**
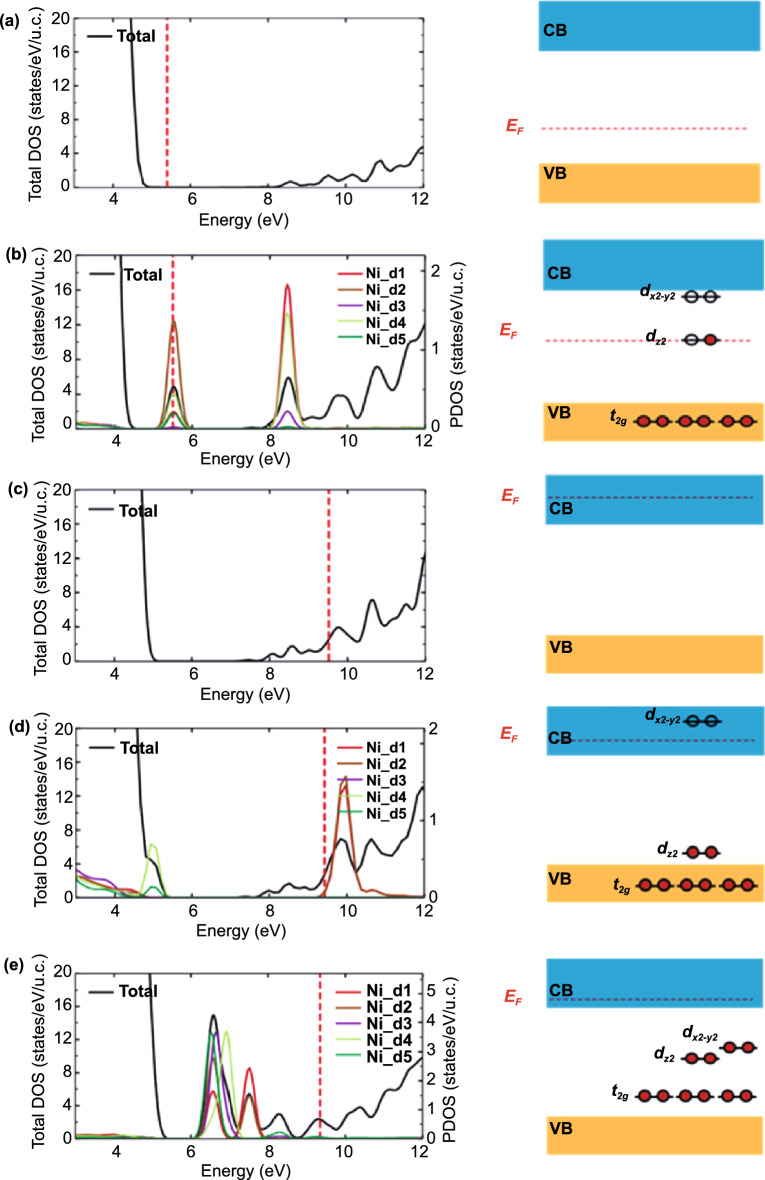
Theoretical calculation. Total density of states (DOS) and projected density of states (PDOS) onto a Ni atom (left panels) and schematics for defect levels (right panels) for **a** pristine In_2_O_3_ and **b** In_2_O_3_ with a substitutional Ni_In_ defect. Total DOS and PDOS onto an Ni atom (left panels) and schematics for defect levels (right panels) for **c** Ref. ITO, **d** ITO with a substitutional Ni_In_ defect, and **e** ITO with an interstitial Ni_In_ defect

To understand the underlying mechanism by which the WF of *m*-TCOs varies, we prototypically investigated the effects of *Ni*-EMi on ITO using density functional theory (DFT) calculations (Fig. 4) (see Experimental Section for details). Based on the EDS and XPS analyses, the crystal structure (or model) of *m*-ITO is given in Fig. S8, wherein metal (i.e., Ni) atoms can substitute In atoms or be interstitially located in the ITO. The generalized gradient approximation (GGA) and the on-site Coulomb correlation (*U)* functional for the exchange–correlation potential were used to estimate a WF of 4.77 eV for pure ITO [23–25], which agrees well with the measured value (Fig. 4a). When Ni atoms substitute for the In atoms in In_2_O_3_, the *e*_*g*_ levels of Ni *d*-orbitals lie in the band gap*,* whereas the inter(non)-axial orbitals arising from the splitting of d-orbital (*t*_*2g*_) levels are located below the valence band maximum (Fig. 4b). At the *e*_*g*_ levels, the *d*_*z2*_ orbital state is half-filled near the Fermi level, and the unfilled *d*_*x2-y2*_ orbital state is located slightly below the conduction band minimum. This indicates that the empty Ni *d* levels can capture electrons, and consequently, the implanted Ni influences the WF of ITO. Because Sn atoms serve as *n*-type dopants, the Fermi level is located above the conduction band minimum (Fig. 4c). The Ni substitutional defect decreases the Fermi level by 0.10 eV (from 9.52 to 9.42 eV) because the half-filled Ni *d*_*z2*_ level captures electrons. This level is subsequently lowered to the valence band edge, as shown in Fig. 4d, whereas the Ni *d*_*x2-y2*_ level remains empty above the minimum conduction band. By contrast, when Ni creates an interstitial defect, the electron capture effect is more significant, filling all Ni *d* levels and thereby lowering the Fermi level by as much as 0.18 eV (Fig. 4e). This result supports the idea that the Fermi level can be lowered by Ni-related defects, where the interstitial Ni elements dominantly increase the WF. On the contrary, the interstitial Cu and Ag atoms elevate the Fermi level of ITO, resulting in a decrease in the WF value, as shown in Fig. S9. This decrease might originate from the relatively lower WF and oxidation energy of pure Cu and Ag compared to the WF of ITO, as analyzed in Fig. 3. Further, this is likely caused by the different electronic configurations of Ag and Cu compared to that of Ni. Metallic Ni has two vacant sites for electrons in the 3*d* orbital, whereas Ag and Cu have fully occupied 3*d* orbitals. As Ni can accept two electrons, it lowers the Fermi level when it is substituted or interstitially doped in the ITO matrix, leading to an increase in the WF value and enforcing the *p*-type property of ITO; by contrast, Ag and Cu prefer to donate an electron to the matrix, thereby elevating the Fermi level and causing the matrix to exhibit more *n*-type characteristics. Consequently, this result elucidates the rationale for the modulation of the WF in TCOs through EMi using various metals.

### Device-Level Validation of *m*-TCOs as Anode or Cathode Electrodes

**Fig. 5:**
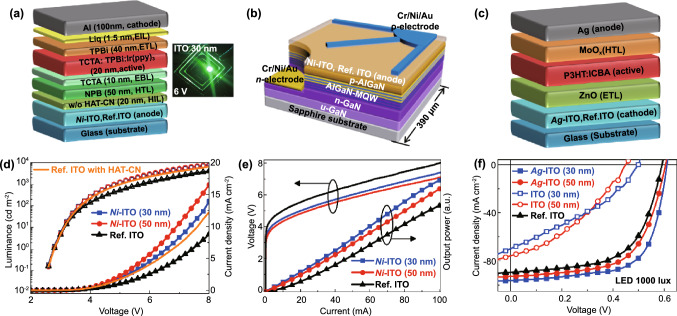
Organic and inorganic devices using *m*-ITOs. Geometry (above) and current (density) versus voltage characteristics (below) of **a, d** OLED, **b, e** inorganic UV LED, and **c, f** OPV

Based on these findings, we employed *m*-ITOs and Ref. ITO as anode (*p*-) or cathode (*n*-) electrodes of various types of optoelectronic devices (i.e., OLED, UV LED, and OPV), which require WF-tunable TCOs, to validate the proposed EMi method at the device level. Initially, we fabricated organic green LEDs and inorganic UV LEDs using *Ni*-ITO as the anode and then inverted OPVs using *Ag*-ITO as the cathode to confirm the versatility of *m*-TCOs. Herein, *Ni*- and *Ag*-ITOs were applied to the devices representatively, due to its superior performance than other *m*-TCOs. Figure 5 shows schematics and exemplary performances of the *Ni*-ITO based (a, d) OLED (*Ni*-ITO/OLED) and (b, e) UV LED (*Ni*-ITO/UV LED), and (c, f) *Ag*-ITO based OPV (*Ag*-ITO/OPV).

#### OLEDs

For the OLED, 30- and 50-nm *Ni*-ITO/OLEDs were fabricated, along with a 150-nm Ref. ITO/OLED for comparison, using a TCTA/TPBi-based green phosphorescent photoactive layer with and without hole injection layer (HIL), as shown in Fig. 5a (see Experimental Section for details). Figure 5d shows the luminance*–*current density*–*voltage curves of the 30- and 50-nm *Ni*-ITO/OLEDs. Compared to Ref. ITO/OLED, both *Ni*-ITO/OLEDs show a higher luminance and current density at driving voltages of 2*–*8 V. Specifically, the driving voltage of both the 30- and 50-nm *Ni*-ITO/OLEDs at 1000 cd/m is ~ 4.9 V, which is reduced by 15% compared to that of Ref. ITO/OLED. Moreover, the current density in the 30- and 50-nm *Ni*-ITO/OLED at 6 V is ~ 79% and 123% greater, respectively, compared to that in Ref. ITO/OLED. We also measured leakage currents for a reverse voltage sweep from 0 to −5 V. Compared to Ref. ITO/OLED, both *Ni*-ITO/OLEDs show much lower leakage currents (Fig. S10a), probably due to the reduced surface roughness of thin *Ni*-ITO films (Fig. S2), demonstrating the reliability of *m*-TCOs during device operation. A higher current density obtained for the 50-nm *Ni*-ITO/OLED over 30-nm *Ni*-ITO/OLED is attributed to the lower *R*_SH_ and higher WF of the 50-nm *Ni*-ITO (Tables S1 and S3). In addition, all luminescence properties (i.e., EL intensity, PE, CE, EQE) of *Ni*-ITO/OLEDs are found to be superior to those of Ref. ITO/OLED (Fig. S10b-d). In particular, the 30-nm *Ni*-ITO/OLED exhibits a higher luminescence efficiency than the 50-nm *Ni*-ITO/OLED owing to the higher transmittance (or light outcoupling) of 30-nm *Ni*-ITO, as discussed earlier. All device performances of the ITO/OLEDs are summarized in Table S4. Consequently, the improved performance of *Ni*-ITO/OLED over Ref. ITO/OLED is attributed in part to the increased WF of *Ni*-ITO, which results in a well-aligned energy level between *Ni*-ITO and the NPB-based hole transport layer, as shown in Fig. 3d. The energy-level alignment reduces the hole injection energy barrier, leading to a minimal potential loss and contact resistance. Furthermore, the high transmittance and the relatively low *R*_SH_ of the 30- and 50-nm *Ni*-ITOs contribute to maximizing the light extraction and minimizing the electrical loss. Notably, *Ni*-ITO/OLEDs without HILs shows better performances than Ref. ITO/OLED with a hexaazatriphenylenehexacabonitrile (HAT-CN) HIL (Figs. 5d and S10), which highlights the superiority of the proposed EMi method in organic-based optoelectronic devices.

#### UV LEDs

Dopant-tunable ultrathin TCOs are also required for UV LEDs. For the UV LED, inorganic AlGaN is used as photoactive and *p*-contact layers (Fig. 5b). The direct ohmic contact with the *p*-AlGaN layer is important owing to the charge injection imbalance caused by the large WF difference between the anode and *p*-AlGaN layers [44]. When 30- and 50-nm *Ni*-ITOs are used as an anode of the UV LED with a *p*-AlGaN contact layer, band alignments between layers can be improved owing to the increased WF of *Ni*-ITO from ~ 4.7 to ~ 5.2 eV. Figure 5e shows the light output power*–*current–voltage curves of the three UV LEDs plotted up to 100 mA. 150-nm Ref. ITO/UV LED shows the forward voltages of 5.5 V (at 20 mA), whereas the 30- and 50-nm *Ni*-ITO/UV LEDs show forward voltages of 5.0 and 4.85 V, respectively. Note that the current injection efficiency into the *p*-Al_0.1_Ga_0.9_ N layer is determined by the electrical properties of the high WF *Ni*- ITO. We also observed the output power increase of 30.9% and 23.3% in the 30- and 50-nm *Ni*-ITO/UV LEDs, respectively, compared to the Ref. ITO/UV LED. EL intensities of the 30- and 50-nm *Ni*-ITO/UV LEDs increase by 30.6% and 22.8%, respectively, compared to that of Ref. ITO/UV LED, as shown in Fig. S11a. The improved light output power and EL intensities are thought to result from much higher transmittance of the 30- and 50-nm *Ni*-ITOs in the UV range (89%–93% at 365 nm), compared to that of 150-nm Ref. ITO (~ 70% at 365 nm). The observed light emission distributions from the 30- and 50-nm *Ni*-ITO/UV LEDs at low (20 mA) and high (50 mA) currents are brighter than the emission from Ref. ITO/UV LED, as shown in Fig. S11b. Compared to Ref. ITO/UV LED, more uniform emission profiles are observed for *Ni*-ITO/UV LEDs at low injection currents, indicating that *Ni*-ITO is more effective than Ref. ITO in terms of current spreading (or distribution) over the ITO as well as vertical injection. The performances of UV LEDs obtained herein are summarized in Table S5. This performance enhancement is interpreted analogically to that in *Ni*-ITO/OLEDs.

#### Indoor OPVs

Lastly, in order to further extend the applicability of *m*-TCOs, we employed *Ag*-ITOs for OPVs, improving energy level alignment (Fig. 3a, e). In this application, we used *Ag*-ITO as the cathode (*i.e.*, the electron-collecting electrode) for indoor OPVs to fully utilize its unique features such as ultrahigh transmittance, WF tunability, and low surface roughness. In particular, it is important to maximize light absorption and minimize current loss to achieve a high-power conversion efficiency (PCE) for indoor OPV because of the limited number of incident photons under indoor lighting [45]. Herein, *Ag*-ITO with a transmittance of 95% at 550 nm and WF ranges of 4.24–4.3 eV was applied as the cathode of inverted OPV, to confirm its validity, as shown in Fig. 5c. Figure 5f shows current density*–*voltage curves of *Ag*-ITO/OPVs and Ref. ITO/OPVs. Compared to 150-nm Ref. ITO/OPV, 30- and 50-nm *Ag*-ITO/OPVs show higher PCE owing to the increased *V*_oc_ and *J*_sc._ The increased *V*_oc_ of *Ag*-ITO/OPVs is attributed to smaller WF values of *Ag*-ITO compared to that of the Ref. ITO according to metal–insulator–metal model [46]. The improved *J*_sc_ in *Ag*-ITO/OPVs can be explained by the synergetic effect of higher transmittance (Fig. S4a) and lower WF (Fig. 3a) of *Ag*-ITO compared to Ref. ITO. Higher transmittance allows more photons to be absorbed in the photoactive layer, generating more charge carriers. In addition, lower WF of *Ag*-ITO helps to facilitate electron collection and transport via band alignments (Fig. 3e). To highlight the superiority of *Ag*-ITO/OPVs, the results of 30- and 50-nm pure (without EMi) ITO/OPVs are also plotted in Fig. 5f. These devices exhibit s-shaped current density–voltage characteristics, with reduced *V*_oc_ and *J*_sc_, probably due to the insufficient charge transport at the contact layer with ultrathin pure ITOs. The 30- and 50-nm pure ITOs show high resistivity and WF misalignment with ZnO electron transport layer (Tables S1*–*S3 and Fig. 3e). The photovoltaic parameters of all OPVs are summarized in Table S6.

## Conclusion

In this study, we proposed novel *m*-TCOs via electric field-driven metal implantation, through which the WF of ultrathin TCOs could be tailored by as much as 0.97 eV without losses in the surface morphology, crystal structure, and electrical and optical properties. Using these *m*-TCOs (e.g., *Ni*- and *Ag*-ITOs) as anode or cathode electrodes, we achieved outstanding performance in both organic and inorganic LEDs as well as organic solar cells to verify the universality of the proposed EMi method. These improvements were attributed to the well-aligned energy band at the interface between *m*-TCO and organic/inorganic semiconductor materials, in addition to the ultrahigh transparency in the UV and visible range, low *R*_SH_, and low surface roughness of *m*-TCO. This study demonstrates that the proposed *m*-TCOs can provide a facile and universal solution to the contradiction between transparency and conductivity in ultrathin film-based transparent electrodes, along with full insight into further improvements in state-of-the-art energy conversion devices.

## Supplementary Information

Below is the link to the electronic supplementary material.

Supplementary file1 (PDF 1302 kb)
