# Supplementary material for: Dopant-Tunable Ultrathin Transparent Conductive Oxides for Efficient Energy Conversion Devices
Source: Nanomicro Lett. 2021 Oct 16;13:211. doi: 10.1007/s40820-021-00735-y (PMC8520554; doi:10.1007/s40820-021-00735-y)
Supplement: Supplementary file 1 — Supplementary file1 (PDF 1302 kb) [file 40820_2021_735_MOESM1_ESM.pdf]

Supporting Information for

Dopant-Tunable Ultra-Thin Transparent Conductive Oxides for Efficient Energy Conversion Devices

Dae Yun Kang[1, #], Bo-Hyun Kim[2, #], Tae Ho Lee[1], Jae Won Shim[1], Sungmin Kim[1], Ha-Jun Sung[3], Kee Joo Chang[3], Tae Geun Kim[1, *]

[1]School of Electrical Engineering, Korea University, Seoul 02841, Republic of Korea

[2]Department of Advanced Materials Engineering, Kongju National University, Cheonan 31080, Republic of Korea

[3]Department of Physics, Korea Advanced Institute of Science and Technology, Daejeon 34141, Republic of Korea

[#]Dae Yun Kang and Bo-Hyun Kim contributed equally to this work

*Corresponding author. E-mail: tgkim1@korea.ac.kr (Tae Geun Kim)

Supplementary Figures and Tables

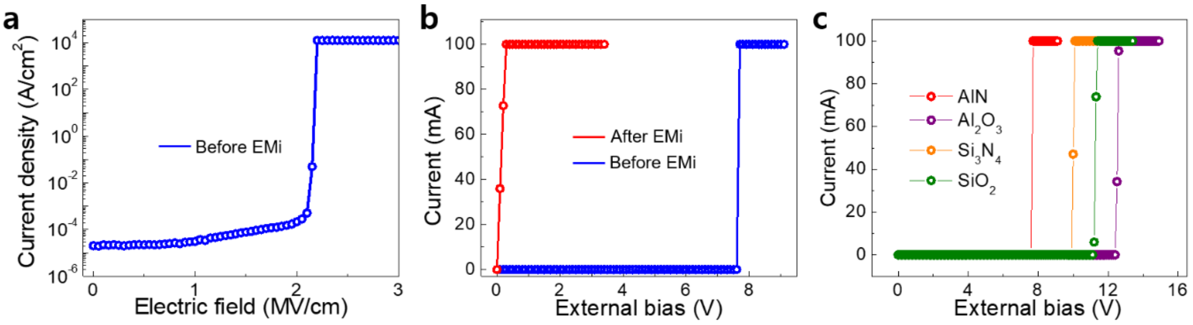

Fig. S1 a Current density versus electric field curve of the Ni/AlN/ITO structure during the EMi process. **b** Current versus voltage properties of the Ni/AlN/ITO structure before and after EMi. **c** Current–voltage characteristics measured for 10-nm AlN, Al_2O_3, Si_3N_4, and SiO_2 buffer layers during the EMi process

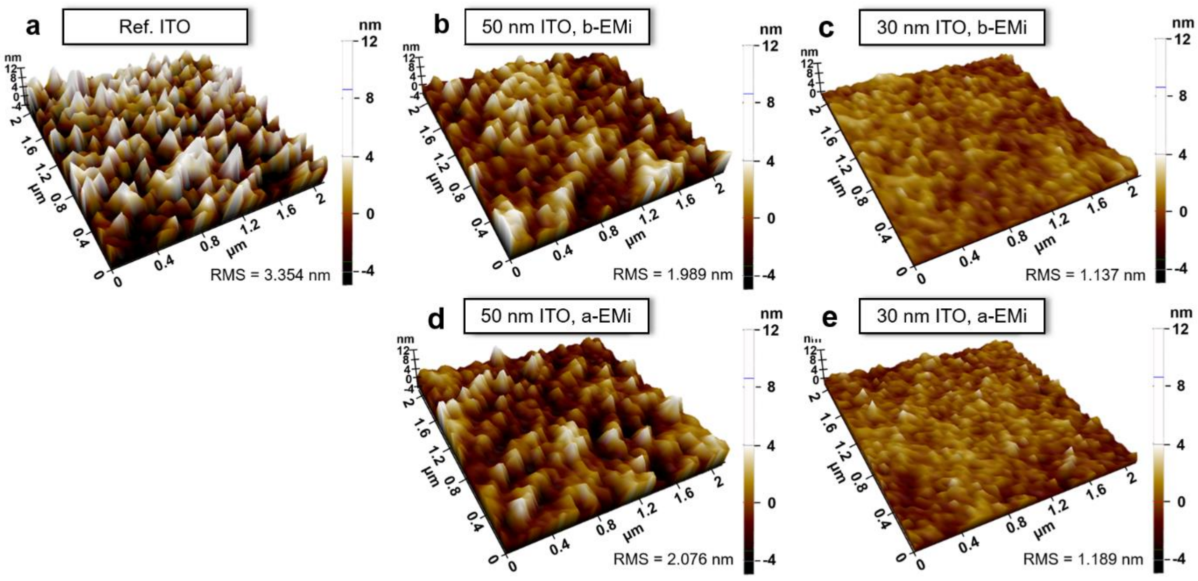

Fig. S2 AFM surface morphologies of **a** 150-nm Ref. ITO, **b** 50- and **c** 30-nm pure ITO films before EMi (b-EMi), and **d** 50- and **e** 30-nm *Ni*-ITO films after EMi (a-EMi). The vertical scale is displayed by the color scale bar

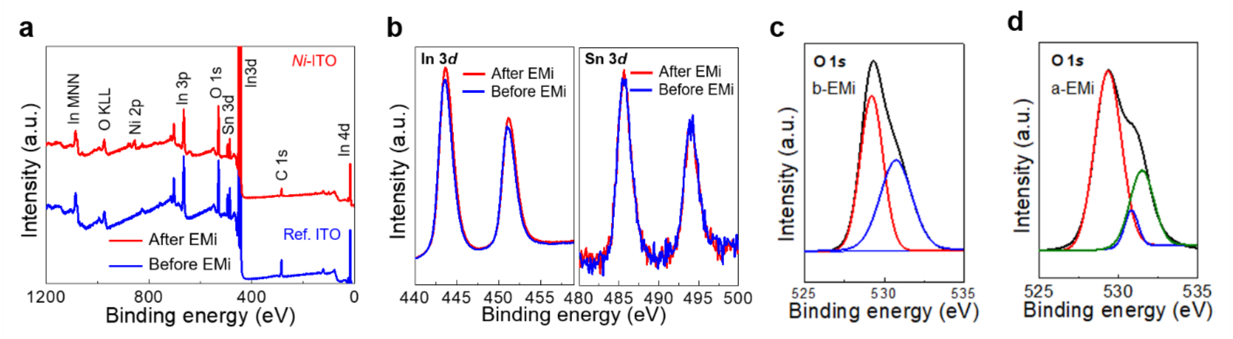

Fig. S3 XPS spectra of ITO before and after EMi for **a** wide scan and **b** In 3*d* and Sn 3*d* core levels. XPS core-level spectra of O 1*s* **c** before *Ni*-EMi and **d** after *Ni*-EMi

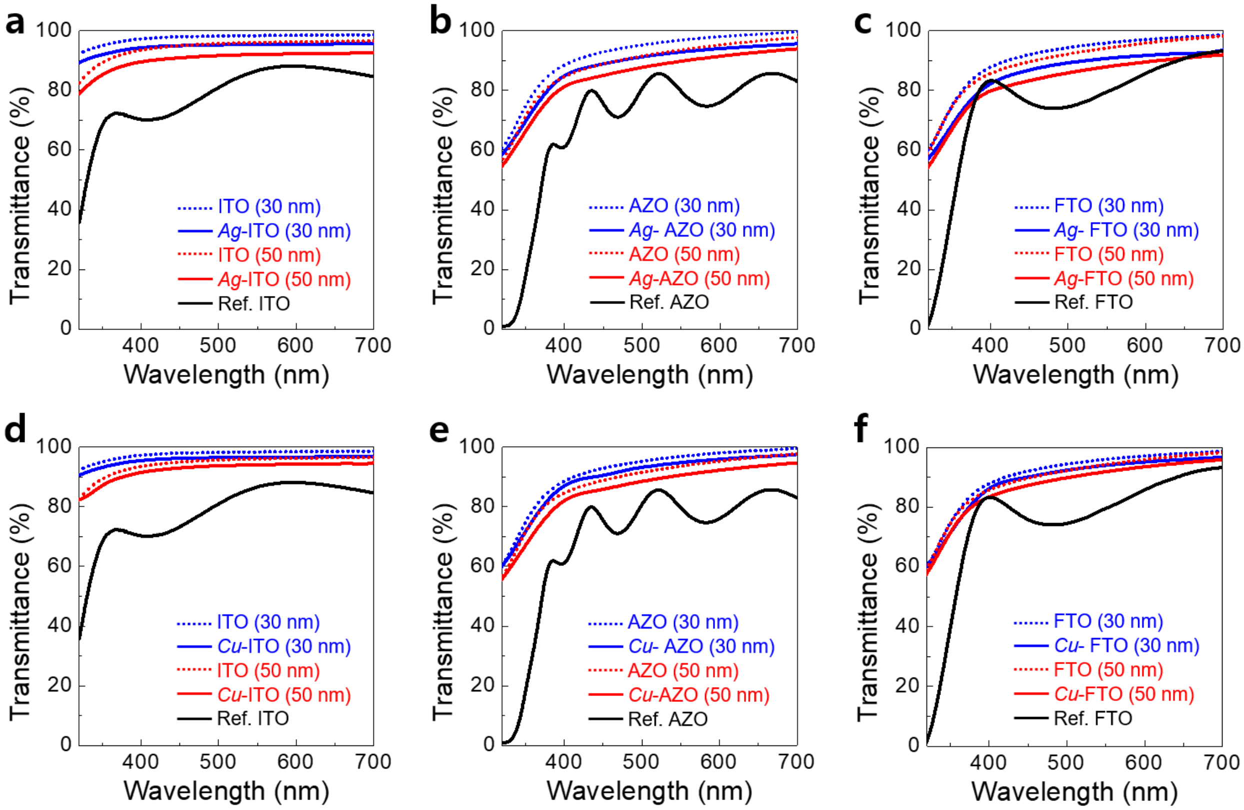

Fig. S4 Comparison of UV–Vis transparency of ITO, AZO, and FTO, respectively, before and after EMi with **a**–**c** Ag and **d**–**f** Cu

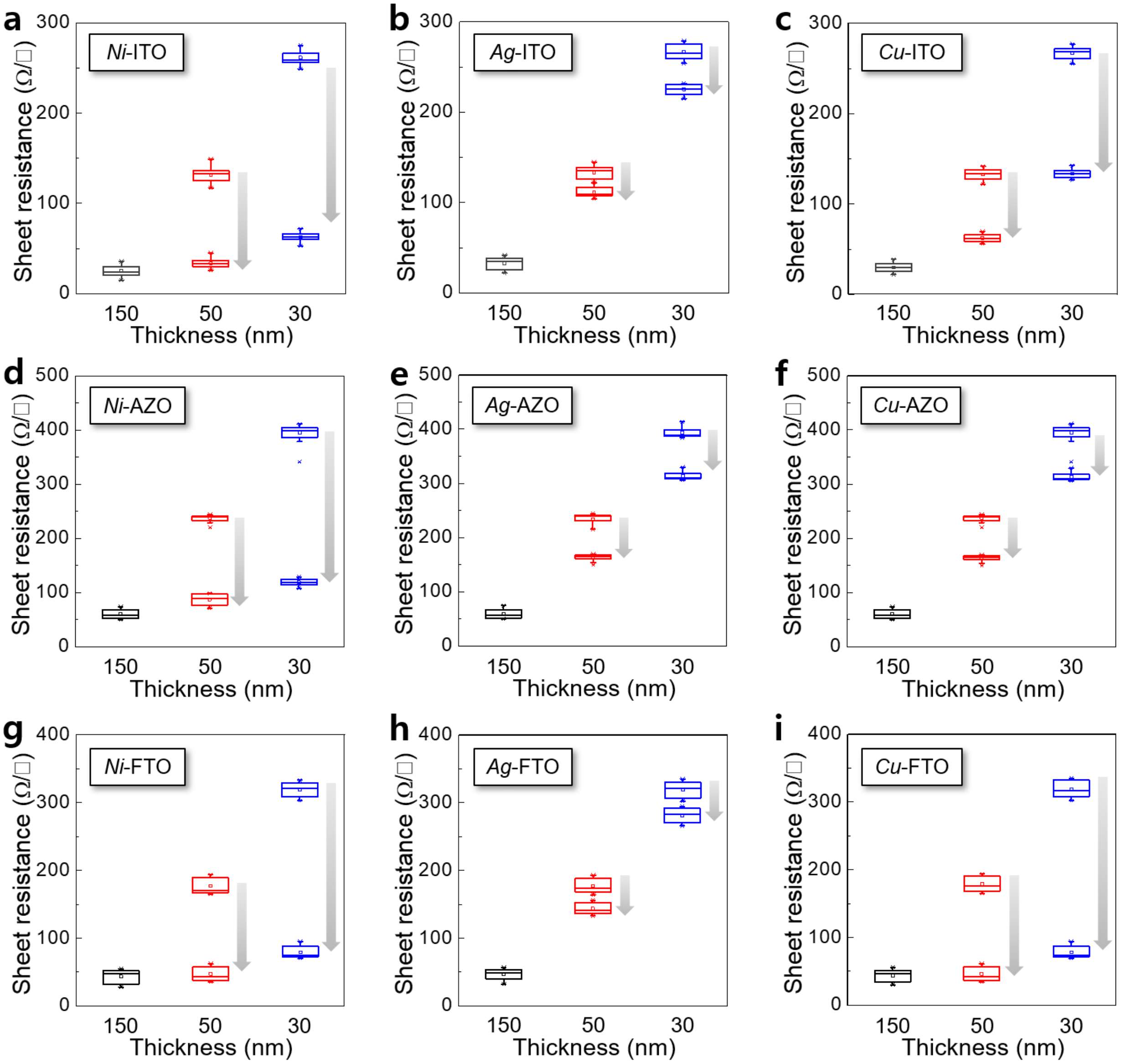

Fig. S5 Comparison of doped metal and film-thickness-dependent sheet resistance: **a–c** *Ni*-, *Ag*- and *Cu*-ITO **d–f** *Ni*-, *Ag*- and *Cu*-AZO, and **g–i** *Ni*-, *Ag*- and *Cu*-FTO. The film thickness of the TCOs decreased from 150 nm to 50 and 30 nm

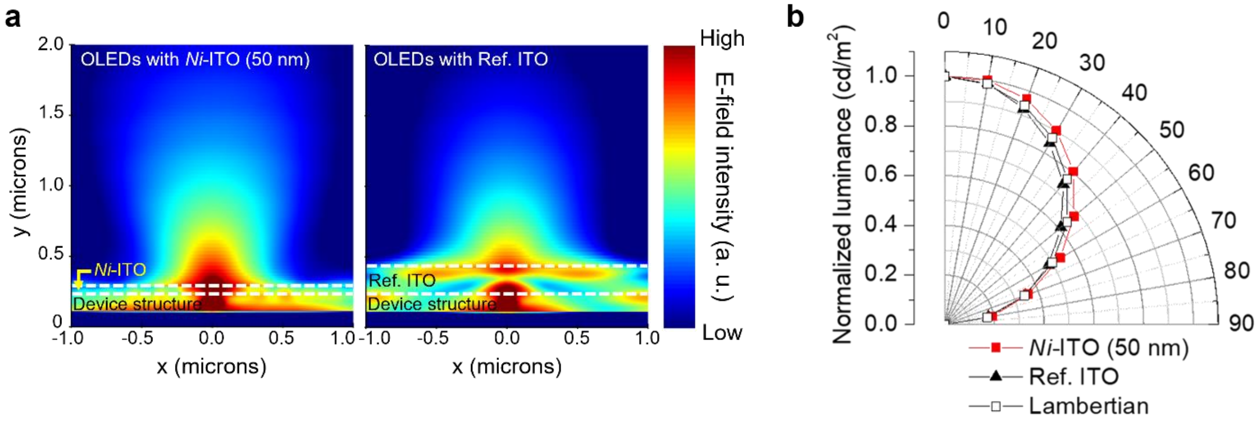

Fig. S6 a Near-field distribution of the OLEDs with 50-nm *Ni*-ITO (left) and 150-nm Ref. ITO (right) anodes. **b** Normalized angular-dependent luminance profiles measured at 7 V for *Ni*-ITO/OLED and Ref. ITO/OLED. The calculated Lambertian profiles are also provided for comparison

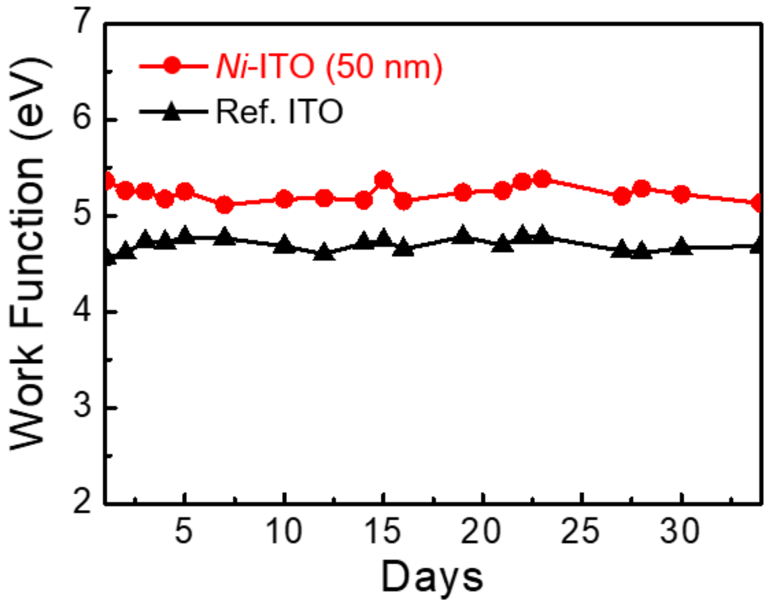

Fig. S7 Work function changes in air over 34 days for the Ref. ITO (black triangle) and 50 nm *Ni*-ITO (red circle). In each case, the values are the averages measured from 10 different points of the film

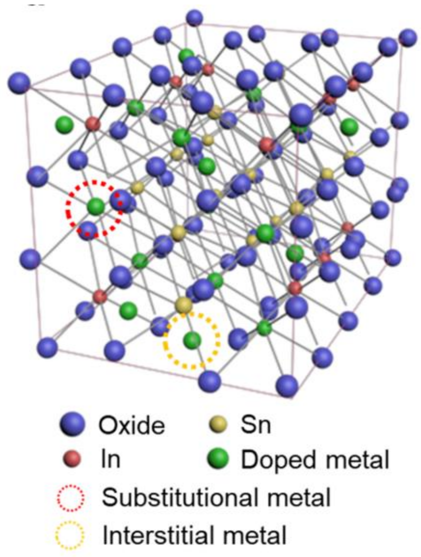

Fig. S8 Ideal atomic structure of the *m*-ITO film with substitutional (red circle) and interstitial (yellow circle) metal impurities

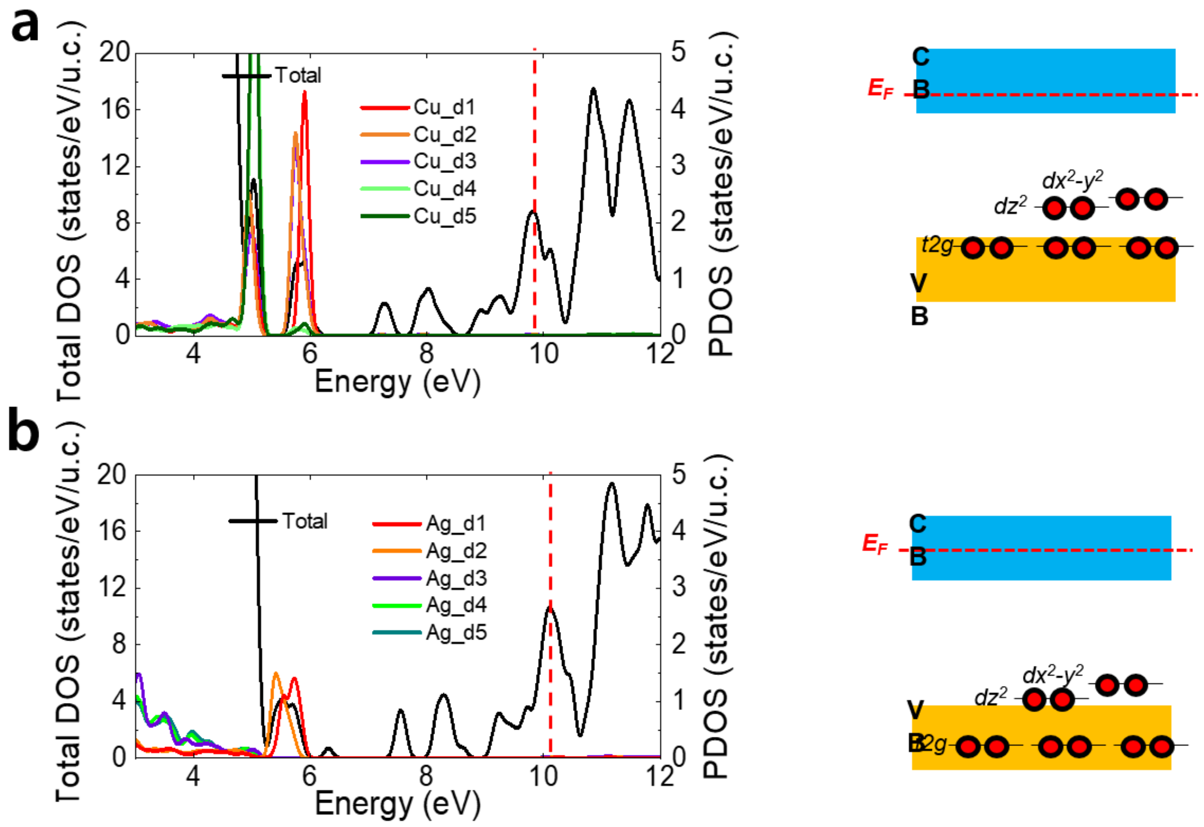

Fig. S9 Theoretical calculation: Total DOS and PDOS corresponding to **a** Cu and **b** Ag atoms (left panels) and schematics of defect levels (right panels) for interstitially positioned Cu and Ag in the ITO matrix

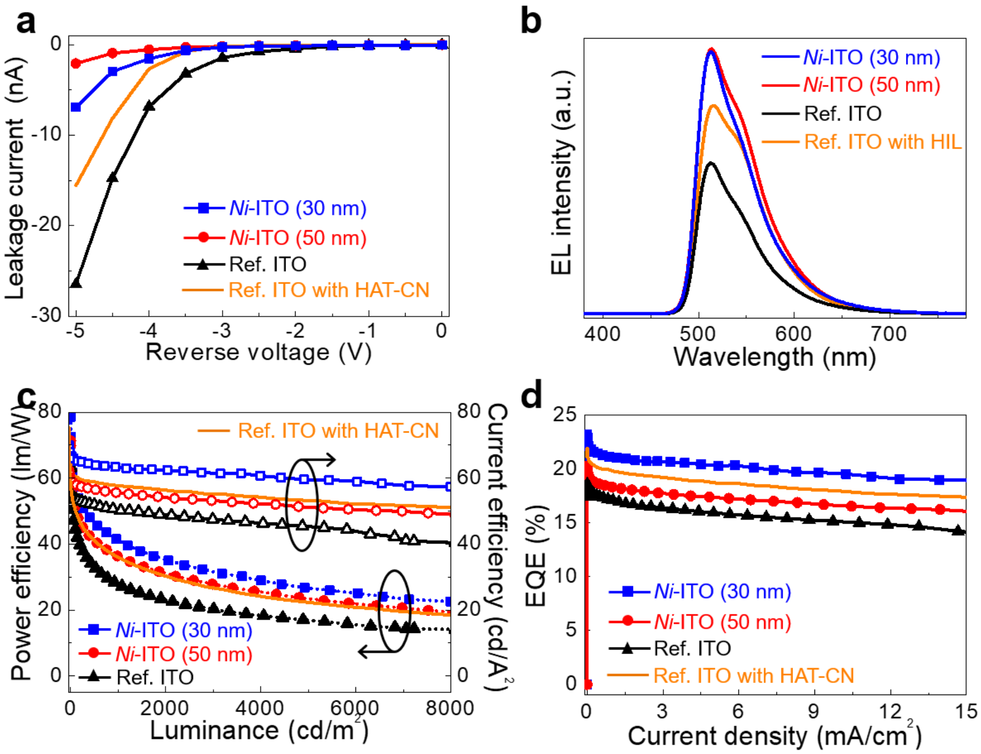

Fig. S10 Leakage currents and luminescence properties (at 5,000 cd/m^2) of green OLEDs (150-nm Ref. ITO/OLED and 30- and 50-nm *Ni*-ITO/OLEDs) with and without hexaazatriphenylene hexacarbonitrile (HAT-CN) HIL. **a** Leakage current characteristics for each OLED. **b** EL intensity spectra versus wavelength for each OLED. **c** Power efficiency (solid symbol) and current efficiency (open symbol) versus luminance for each OLED. **d** External quantum efficiency versus current density for each OLED

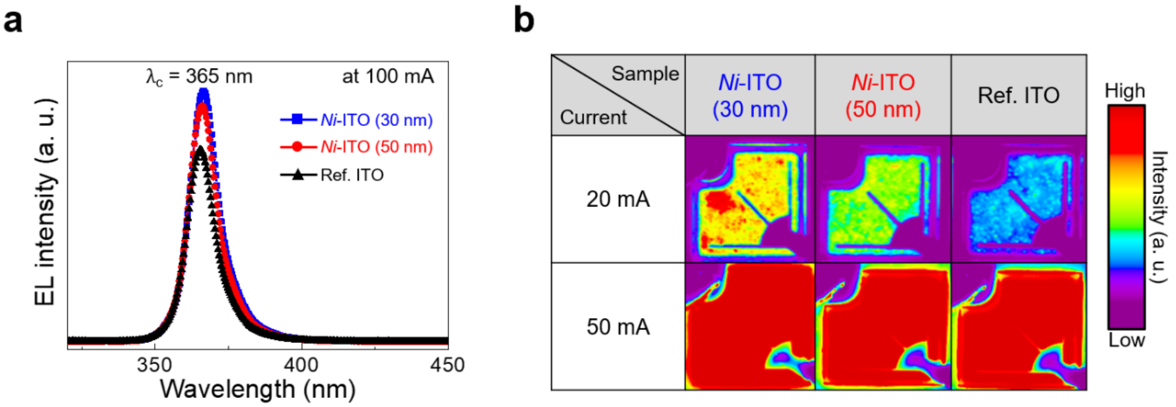

Fig. S11 a Electroluminescence spectra as a function of wavelength measured for each sample at 100 mA. **b** Microscopic light emission photographs measured at 20 mA and 50 mA for each sample. The linear color scale is the relative intensity of the emission distribution

Table S1 Sheet resistance (R_{SH}), resistivity (ρ), and transmittance (T) of ITO, AZO, and FTO films after *Ni*-EMi process. In each case, the values are averages measured from 10 specimens

Electrodes	Thickness	30 nm		50 nm		150 nm
	Ni-EMi	Before	After	Before	After	Ref. TCO
ITO	Sheet resistance, R_{SH} (Ω/□)	261 ± 15	61 ± 10	13 ± 18	34 ± 11	26 ± 11
	Resistivity, ρ (10^{-4} Ω·cm)	7.83 ± 0.4	1.83 ± 0.3	6.55 ± 0.9	1.71 ± 0.5	3.90 ± 1.6
	Transmittance at 550 nm, T (%)	98.4 ± 0.2	96.8 ± 0.1	96.1 ± 0.3	93.9 ± 0.1	86.7 ± 0.3
AZO	Sheet resistance, R_{SH} (Ω/□)	398 ± 17	151 ± 12	228 ± 15	87 ± 18	61 ± 13
	Resistivity, ρ (10^{-4} Ω·cm)	11.94 ± 0.5	4.53 ± 0.3	11.40 ± 0.7	4.35 ± 0.8	9.15 ± 1.9
	Transmittance, T at 550 nm (%)	96.9 ± 0.3	95.5 ± 0.1	93.6 ± 0.3	91.5 ± 0.1	79.7 ± 0.5
FTO	Sheet resistance, R_{SH} (Ω/□)	317 ± 17	81 ± 14	177 ± 18	47 ± 15	42 ± 14
	Resistivity, ρ (10^{-4} Ω·cm)	9.51 ± 0.4	2.43 ± 0.3	8.85 ± 0.8	2.35 ± 0.7	6.30 ± 2.1
	Transmittance, T at 550 nm (%)	96.0 ± 0.3	93.5 ± 0.3	94.3 ± 0.2	90.9 ± 0.1	79.5 ± 0.4

Table S2 Carrier density and hole mobility of ITO, AZO, and FTO films after the *Ni*-EMi process

Electrode	Thickness	30 nm		50 nm		150 nm
	Ni-EMi	Before	After	Before	After	Ref. TCO
ITO	Carrier density (10^{21} cm^{3})	0.649 ± 0.04	1.311 ± 0.08	0.693 ± 0.11	1.319 ± 0.16	0.871 ± 0.18
	Mobility (cm^2/V·s)	11.8 ± 1.2	26.1 ± 2.3	13.8 ± 2.7	27.9 ± 2.6	18.4 ± 2.1
AZO	Carrier density (10^{21} cm^{3})	0.51 ± 0.04	0.899 ± 0.03	0.538 ± 0.04	1.109 ± 0.03	0.558 ± 0.04
	Mobility (cm^2/V·s)	10.3 ± 1.1	15.3 ± 1.3	10.2 ± 1.5	13.0 ± 1.7	12.24 ± 1.9
FTO	Carrier density (10^{21} cm^{3})	0.555 ± 0.03	1.137 ± 0.08	0.587 ± 0.05	1.311 ± 0.13	0.693 ± 0.17
	Mobility (cm^2/V·s)	11.8 ± 1.1	22.6 ± 0.3	12.0 ± 0.2	20.3 ± 0.1	14.3 ± 0.4

Table S3 Work function values of 30-, 50- (before EMi), and 150-nm Ref. ITO, AZO, and FTO films, and Ni-implanted (after EMi) 30- and 50-nm ITO, AZO, and FTO films. Empty cells indicate no measurement for the corresponding films. The values are the averages measured from three different specimens for the UV photoelectron spectroscopy (UPS), and 10 different points (for 3 samples) for the KP, respectively.

Electrodes		Work function (eV)			
		UPS		Kelvin probe in air	
		Before EMi	After EMi	Before EMi	After EMi
ITO	30 nm	4.77 ± 0.03	5.09 ± 0.04	4.61 ± 0.02	5.04 ± 0.03
	50 nm	4.79 ± 0.02	5.27 ± 0.03	4.64 ± 0.04	5.17 ± 0.10
	Ref. (150 nm)	4.82 ± 0.03	—	4.67 ± 0.05	—
AZO	30 nm	4.58 ± 0.03	5.04 ± 0.03	4.69 ± 0.03	5.15 ± 0.03
	50 nm	4.60 ± 0.01	5.16 ± 0.05	4.70 ± 0.08	5.26 ± 0.05
	Ref. (150 nm)	4.65 ± 0.02	—	4.72 ± 0.05	—
FTO	30 nm	—	—	4.81 ± 0.04	5.17 ± 0.03
	50 nm	—	—	4.89 ± 0.02	5.31 ± 0.05
	Ref. (150 nm)	—	—	4.91 ± 0.03	—

Table S4 Device performance of OLEDs including the driving voltage (V_D), current efficiency (CE), power efficiency (PE), and external quantum efficiency (EQE). In each case, the values are the averages measured from 10 different specimens

Sample name	HIL (HAT-CN)	V_D at 1000 cd/m^2 (V)	CE at 1000 cd/m^2 (cd/A)	PE at 1000 cd/m^2 (lm/W)	Max. EQE (%)
Ni-ITO (30 nm)	Without HIL	4.83 ± 0.1 (15.3%↓)	63.5 ± 1.2 (26.2%↑)	41.3 ± 0.7 (49.1%↑)	23.2 ± 0.3 (24.7%↑)
Ni-ITO (50 nm)		4.81 ± 0.1 (15.6%↓)	55.2 ± 1.3 (9.7%↑)	35.9 ± 0.9 (29.6%↑)	20.3 ± 0.2 (9.1%↑)
Ref. ITO (150 nm)		5.7 ± 0.1	50.3 ± 1.1	27.7 ± 1.0	18.6 ± 0.4
Ref. ITO (150 nm)	With HIL	5.05 ± 0.13 (11.4%↓)	60.1 ± 1.5 (19.5%↑)	39.5 ± 1.2 (42.6%↑)	22.0 ± 0.3 (18.3%↑)

Table S5 Device performance of 365-nm UV LEDs including the forward voltage (V_F), reverse leakage current (I_L), and light output power (P_O). In each case, the values are the averages measured from 10 different specimens

Sample name	V_F at 20 mA (V)	I_L at 5 V (nA)	P_O at 100 mA (a.u.)
Ni-ITO (50 nm)	4.85 ± 0.1 (12%↓)	-19.2 ± 2.6 (51%↓)	53.4 ± 1.1 (23%↑)
Ni-ITO (30 nm)	5.0 ± 0.1 (9%↓)	-27.6 ± 3.1 (30%↓)	56.7 ± 1.3 (31%↑)
Ref. ITO (150 nm)	5.5 ± 0.1	-39.4 ± 3.3	43.3 ± 1.7

Table S6 Device performance of OPVs including the open-circuit voltage (V_{OC}), short-circuit current (J_{SC}), fill factor (FF) and power conversion efficiency (PCE). In each case, the values are the averages measured from 10 different specimens

**Sample Name**	V_{OC} **(mV)**	J_{SC} **(μA cm^{-2})**	FF **(%)**	PCE **(%)**
Ag-ITO (30 nm)	611 ± 3	96.1 ± 1.1	65.8 ± 1.3	13.8 ± 0.3
Ag-ITO (50 nm)	605 ± 1	92.7 ± 2.2	61.3 ± 1.6	12.1 ± 0.2
Pure ITO (30 nm)	499 ± 11	69.9 ± 0.5	34.0 ± 2.7	4.2 ± 0.7
Pure ITO (50 nm)	450 ± 13	75.2 ± 0.7	38.1 ± 2.4	4.8 ± 0.5
Ref. ITO (150 nm)	592 ± 1	89.5 ± 2.9	60.2 ± 0.5	11.4 ± 0.5
